# Euthanasia and assisted suicide of patients with psychiatric disorders in the Netherlands between 2021 and 2023

**DOI:** 10.1192/j.eurpsy.2025.723

**Published:** 2025-08-26

**Authors:** M. Lucente, P. Pietrini, I. A. Cristea

**Affiliations:** 1 General Psychology, University of Padua, Padua; 2 IMT Scuola Alti Studi Lucca, Lucca, Italy

## Abstract

**Introduction:**

Medical-assistance in dying, either by euthanasia or assisted suicide (EAS), for mental disorders as the main reason is a complex and controversial practice. owing to the inevitable tension between the seriousness of the mental disorder and the requirement that the patient can make a well-reasoned decision. Systematic investigations of the characteristics of the patients (disorders, previous treatment, comorbidities) and the procedures of granting EAS for mental disorders have been limited.

**Objectives:**

To describe the characteristics of patients receiving EAS for psychiatric conditions in the Netherlands between 2021 and 2023.

**Methods:**

We reviewed psychiatric EAS case summaries made available publicly online by the Dutch regional euthanasia review committees between 2021 and 2023. All summaries were translated with ChatGPT and those with mental disorders as the main reason were selected. We extracted information about the patients’ clinical and social characteristics, type of treatments previously administered, how physicians handled patients’ requests and the euthanasia review committees’ assessments of the physicians’ actions.

**Results:**

Thirty-six cases were identified. Of these, 86% (n = 31) were women. Regarding age, 30.5% (n = 11) were 70 years or older, 36% (n = 13) were 50 to 70 years old, 16.6% (n = 6) were 30 to 50 years old and 16.6% (n = 6) were less than 30 years old. Most had chronic conditions, with histories of attempted suicides (39%; n=14) and psychiatric hospitalizations (69%; n=25). One third (n = 12) of patients were described as socially isolated. Depressive disorders were the primary psychiatric diagnosis in 27.7% (n = 10) of cases. Other conditions included personality disorders (19.4%; n=7), particularly borderline (11%; n=4), somatoform disorders (17%; n=6) and posttraumatic stress disorder (11%; n=4). Comorbidities with medical conditions or physical symptoms were present in 27.7% (n = 10) of cases. Most frequent treatments previously administered were medications and psychotherapy, particularly cognitive behavioral (19.4%; n=7) therapy and EMDR (14%; n=5). Fifty percent (n = 18) of patients received EAS from Euthanasia Expertise Center physicians, 17% (n=6) from their general practitioner. In 11% (n = 4) of the cases there was disagreement among consultants.

**Conclusions:**

Individuals who received EAS for psychiatric reasons in the Netherlands between 2021 and 2023 were mostly women, with complex and chronic psychiatric conditions, often with medical comorbidities. Despite the fact that EAS requires agreement from two independent physicians, there were instances where it was performed without it.

**Disclosure of Interest:**

None Declared

